# Elevated plasma levels of Krebs von den Lungen-6 and geographic appearance on high-resolution computed tomography are associated with diffuse alveolar damage in autopsy cases of acute respiratory distress syndrome: a retrospective study

**DOI:** 10.1186/s12890-022-02102-y

**Published:** 2022-08-11

**Authors:** Ryosuke Imai, Daisuke Yamada, Yutaka Tomishima, Tomoaki Nakamura, Clara So, Shosei Ro, Kohei Okafuji, Atsushi Kitamura, Torahiko Jinta, Naoki Nishimura

**Affiliations:** 1 Department of Pulmonary Medicine, Thoracic Center, St. Luke’s International Hospital, 9-1, Akashi-cho, Chuo City, Tokyo, 104-8560 Japan; 2 Department of Radiology, St. Luke’s International Hospital, Tokyo, Japan

**Keywords:** Acute respiratory distress syndrome, Chest high-resolution computed tomography, Diffuse alveolar damage, Geographic appearance, KL-6

## Abstract

**Background:**

Although diffuse alveolar damage (DAD) is a histopathological hallmark of acute respiratory distress syndrome (ARDS), its detection without lung biopsy is challenging. In patients with ARDS, the specificity of the Berlin definition to diagnose DAD as a reference standard is not adequately high, making it difficult to adequately diagnose DAD. The purpose of this study was to investigate the relationship between DAD and clinical findings, including KL-6 and geographic appearance, in ARDS patients and to identify more specific diagnostic criteria for DAD.

**Methods:**

Among all adult autopsy cases at a tertiary hospital in Japan between January 2006 and March 2021, patients with ARDS who met the Berlin definition criteria were included. The patients’ conditions were classified according to histopathological patterns as DAD or non-DAD, and clinical characteristics, laboratory data, and high-resolution computed tomography (HRCT) findings were compared between the two groups.

**Results:**

During the study period, 27 met the Berlin definition (median age: 79 years, 19 men), of whom 18 (67%) had DAD and 9 (33%) did not. In the non-DAD group, histopathologic findings revealed organizing pneumonia in seven patients and pulmonary hemorrhage in two patients. On HRCT at onset, patients with DAD had more geographic appearance than those without DAD (89% vs. 44%). In patients with geographic appearance and elevated KL-6 (> 500 U/mL), the sensitivity and specificity for DAD diagnosis were 56% and 100%, respectively. All three patients with no geographic appearance and normal KL-6 did not have DAD.

**Conclusions:**

Geographic appearance on HRCT combined with KL-6 levels may predict the presence of DAD in patients with ARDS.

## Background

Acute respiratory distress syndrome (ARDS) is characterized by an acute onset of hypoxemia with bilateral pulmonary infiltrates on chest radiography, secondary to underlying disorders associated with lung injury caused by nonspecific hyperinflammation with neutrophils. Diffuse alveolar damage (DAD) is the histopathological hallmark of ARDS [1, 2]. The presence of DAD is challenging to detect without lung biopsy, but this procedure is invasive, and samples are usually not obtained in patients with ARDS.

The Berlin definition has traditionally been used as a criterion to clinically define ARDS. A study analyzing 712 autopsied patients showed that the sensitivity and specificity of the Berlin definition to diagnose DAD were 89% and 63%, respectively [2]. Although the Berlin definition is advantageous for screening due to its high sensitivity, its low specificity means that some of the identified cases follow a clinical course that differs from that of DAD. ARDS patients without DAD present heterogeneous histopathologic findings, including organizing pneumonia and infection [3]. This is one of the reasons why it is difficult to elucidate the molecular mechanisms of ARDS and to develop specific therapies. Noninvasive methods to identify DAD are therefore an unmet need [4].

Previous studies reported that a lower ratio of arterial oxygen partial pressure to fractional inspired oxygen (PaO_2_/FiO_2_), extensive opacities involving the four quadrants, and increased dynamic driving pressure were associated with DAD [2, 5, 6]. However, one study with 258 ARDS patients who underwent open lung biopsy concluded that DAD could not be predicted clinically [7]. Other findings, such as elevated levels of Krebs von den Lungen-6 (KL-6) and geographic appearance on high-resolution computed tomography (HRCT), have also been associated with DAD. Several previous studies have reported that elevated KL-6 levels are associated with ARDS diagnosis in at-risk populations and ARDS-related mortality [8, 9]. Moreover, geographic appearance is currently considered a typical HRCT finding in ARDS cases [10], and a previous study that evaluated autopsy results from ARDS cases revealed these findings to be consistent with the exudative phase of DAD [11]. However, few reports have examined the association between these findings and DAD in detail.

This study aimed to investigate the relationship between DAD and clinical findings, including KL-6 and geographic appearance, in ARDS patients and to identify more specific diagnostic criteria for DAD.

## Methods

### Subjects

Consent for autopsy is routinely requested from relatives of patients who die in our institution. Among all autopsy cases at our hospital in Japan between January 2006 and March 2021, patients with ARDS who met the Berlin definition criteria were included. Eligible patients were adults (≥ 16 years old) and patients who required ventilation, including noninvasive ventilation, for respiratory failure; those with “do not intubate order” were also included. Patients with chronic interstitial lung disease were excluded.

### Data collection

Medical records were reviewed retrospectively to analyze demographic information, underlying illness, primary risk factor of ARDS, laboratory and radiological data, as well as medical treatment and clinical outcomes. For laboratory data, including KL-6, values at diagnosis were reviewed. In our facility, KL-6 is tested in most patients with ARDS to investigate the cause, but not all patients are tested. Missing values are noted in the results section and in the tables.

### HRCT assessment

In our facility, all patients suspected of ARDS underwent whole-lung volumetric HRCT scanning of the chest at diagnosis using a multidetector-row CT scan to rule out chronic interstitial lung disease, infection, pleural effusion, or lung collapse.

**Fig. 1:**
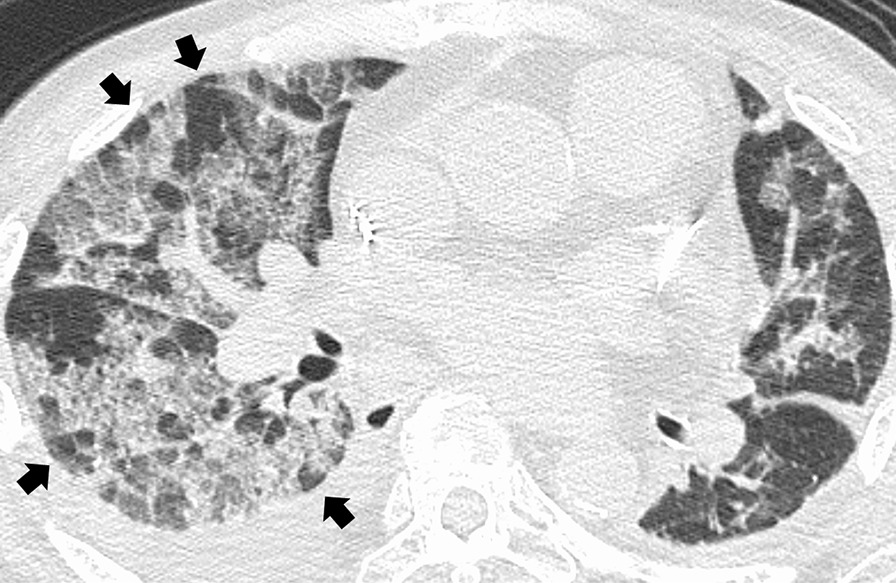
A 70-year-old man, who developed respiratory failure during antibiotic therapy for *Staphylococcus aureus* bacteremia, was diagnosed with acute respiratory distress syndrome. High-resolution CT at diagnosis showed bilateral diffuse reticulation with geographic appearance (black arrows), well-circumscribed infiltrations partially spared in secondary pulmonary lobule units, and bilateral pleural effusions. Although the patient was placed on mechanical ventilation in addition to being administered antibiotics, he passed away 4 days after diagnosis, and autopsy was performed. Pathological findings of the lungs revealed hyaline membrane formation along dilated alveolar ducts and alveoli, with shrinkage of adjacent alveoli and hyperplasia of type II pneumocytes, representing the exudate phase of diffuse alveolar damage

HRCT scans at onset were evaluated by a respiratory specialist and a radiology specialist, blinded to the patients’ clinical characteristics and study outcomes. Disagreements between observers were resolved by consensus. We evaluated the presence of the following specific HRCT findings: ground-glass opacity, consolidation, fibrosis, traction bronchiectasis, honeycombing, and geographic appearance. Geographic appearance was defined as a well-circumscribed infiltration partially spared in secondary pulmonary lobule units and a similar appearance of clear demarcation formed by the coastline against the sea (Fig. 1) [11, 12]. The presence of these findings was assessed qualitatively (either yes or no).

### Pathological assessment

A postmortem study was performed within 24 h of death. After removal from the thorax, the lungs were inflated with 10% formalin and fixed in a block of 10% formalin. After 48 h, the seedlings were cut into slices of 3 cm thickness. The samples for microscopic analysis were obtained from each pulmonary lobe. Patients were classified as having DAD if they had hyaline membranes, even if the membranes were present in only one lobe, as this finding could represent an incipient form of DAD [2].

### Statistical analyses

Data are presented as median (range) for continuous variables and as frequencies (percentage) for categorical variables. Categorical variables were compared using Fisher’s exact test, and continuous variables were compared using the Mann–Whitney U test. All statistical tests were two-tailed, and significance was set at *P* ≤ 0.05. Sensitivity, specificity, and likelihood ratio for identifying ARDS patients with DAD were calculated for all factors associated to a significant *P* value. All analyses were performed using R software, version 3.5.2 (R Foundation for Statistical Computing, Vienna, Austria).

## Results

Of the 767 patients who underwent autopsy during the study period, 27 patients met the Berlin definition criteria and were diagnosed with ARDS prior to death (median age: 79 years, 19 men), of whom 18 (66%) had DAD and 9 (33%) had no DAD. Among the non-DAD group, histopathologic findings revealed organizing pneumonia in seven patients and pulmonary hemorrhage in two patients.

Table 1 presents the clinical characteristics of the patients with and without DAD. The primary causes of ARDS included pneumonia in 10 (56%) and connective tissue disease in 3 patients (17%) with DAD, and pneumonia in 5 (56%) and drug-induced pneumonitis in 2 patients (22%) without DAD. The severity of ARDS at diagnosis was mild in 2 (7%) patients, moderate in 19 (70%) patients, and severe in 6 (22%) patients. One patient of the 27 did not have KL-6 measured at diagnosis. Laboratory data at diagnosis showed that patients with DAD had higher KL-6 levels than those without DAD. The upper limit of normal for KL-6 (500 U/ml) was higher in 11 patients (65%) in the DAD group compared with the 1 (11%) in the non-DAD group. Approximately half of all patients received corticosteroids to treat their ARDS, in most cases in the form of methylprednisolone pulse therapy.

**Table 1:** Demographic and clinical characteristics between patients with DAD and non-DAD

Characteristic	Total (n = 27)	DAD (n = 18)	Non-DAD (n = 9)	*P* value
Age, years	79 (62–92)	79 (62–92)	80 (66–91)	0.757
Male	19 (70)	13 (72)	6 (67)	1.000
BMI	22.1 (16.4–28.8)	21.9 (17.1–28.8)	22.6 (16.4–26.2)	0.471
PaO_2_/FiO_2_	142 (48–258)	142 (48–203)	142 (60–258)	0.877
APACHE II score	29 (18–44)	28 (21–41)	34 (18–44)	0.469
Severity at diagnosis				0.655
Mild	2 (7)	1 (5)	1 (11)	
Moderate	19 (70)	12 (67)	17 (78)	
Severe	6 (22)	5 (28)	1 (11)	
Ventilation				0.683
Noninvasive ventilation	10 (37)	6 (33)	4 (44)	
Mechanical ventilation	17 (63)	12 (67)	5 (56)	
Underlying diseases				
Diabetes mellitus	6 (22)	3 (17)	3 (33)	0.367
Malignance	6 (22)	3 (17)	3 (33)	0.367
Immunosuppression	5 (19)	2 (11)	3 (33)	0.295
End stage renal disease	1 (4)	0 (0)	1 (11)	0.333
Primary risk factor of ARDS				0.916
Pneumonia	15 (56)	10 (56)	5 (56)	
Connective tissue disease	4 (15)	3 (17)	1 (11)	
Drug-induced	3 (11)	1 (6)	2 (22)	
Sepsis	2 (7)	1 (6)	1 (11)	
Malignancy	1 (4)	1 (6)	0 (0)	
Aspiration	1 (4)	1 (6)	0 (0)	
Radiation	1 (4)	1 (6)	0 (0)	
Duration of ARDS before death, d	18 (1–51)	18 (4–51)	18 (1–40)	0.410
Laboratory data at diagnosis				
WBC, per mm^3^	10,300 (300–37,800)	11,200 (300–37,800)	10,300 (2,300–30,000)	0.877
CRP, mg/dL	13.6 (3.5–39.5)	15.3 (3.5–39.5)	11.2 (5.5–21.2)	0.291
Albumin, g/dL	2.5 (1.4–3.3)	2.5 (1.4–3.2)	2.4 (1.4–3.3)	0.570
Creatinine, mg/dL	1.14 (0.27–4.64)	1.13 (0.50–4.56)	1.33 (0.27–4.64)	0.471
LDH, U/L	343 (212–1,192)	425 (212–1,192)	317 (233–705)	0.345
KL-6*, U/mL	413 (194–1,780)	760 (194–1,780)	322 (229–1,322)	0.090
KL-6* > 500 U/mL	12 (44)	11 (65)	1 (11)	0.014
Treatment				
Corticosteroid administration	14 (52)	10 (56)	4 (44)	0.695
Methylprednisolone pulse therapy^†^	13 (48)	10 (56)	3 (33)	0.420
Autopsy findings				
Weight of two lungs, g	1,380 (545–4,605)	1,455 (675–4,605)	1,100 (545–2,375)	0.143
DAD	18 (67)	18 (100)	–	
Organizing pneumonia	7 (26)	–	7 (78)	
Pulmonary hemorrhage	2 (7)	–	2 (22)	

Continuous variables are expressed as medians (ranges), and categorical data are expressed as n (%)

*APACHE* Acute Physiology and Chronic Health Evaluation, *BMI* body mass index, *CRP* C-reactive protein, *DAD* diffuse alveolar damage, *KL-6* Krebs von den Lungen-6, *LDH* lactate dehydrogenase, *PaO*_*2*_*/FiO*_*2*_ arterial oxygen partial pressure/fractional inspired oxygen, *WBC* white blood cell

*Total n = 26; one case of non-DAD had a missing value

^†^ ≥ 500 mg/day for 3 days

Table 2 shows a comparison of HRCT findings between the patients with and without DAD. The patients with DAD had more geographic appearance than those without DAD (16 [89%] vs. 4 [44%], *P* = 0.023). The sensitivity and specificity of the presence of geographic appearance for the diagnosis of DAD were 89% (95% confidence interval [CI] 65–99%) and 56% (95% CI 21–86%), respectively. The sensitivity and specificity for the diagnosis of DAD were examined by combining the KL-6 values that differed the most in proportion between the patients with and without DAD among the laboratory findings. The specificity of geographic appearance and ≥ 500 U/mL KL-6 improved to 100% (95% CI 56–100%) (Table 3).

**Table 2:** HRCT findings at onset between the patients with DAD and non-DAD

HRCT findings	Total (n = 27)	DAD (n = 18)	Non-DAD (n = 9)	*P* value
Ground-glass opacity	27 (100)	18 (100)	9 (100)	NA
Consolidation	19 (70)	14 (78)	5 (56)	0.375
Fibrosis	5 (19)	3 (17)	2 (22)	1.000
Honeycombing	1 (4)	1 (6)	0 (0)	1.000
Traction bronchiectasis	20 (74)	14 (78)	6 (67)	0.653
Volume loss	23 (85)	16 (89)	7 (78)	0.582
Geographic appearance	21 (78)	16 (89)	4 (44)	0.023

Data are expressed as n (%)

*HRCT* high-resolution computed tomography, *DAD* diffuse alveolar damage

**Table 3:** Sensitivity and specificity of KL-6 and geographic appearance for the diagnosis of DAD

	Sensitivity, % (95% CI)	Specificity, % (95% CI)	Positive likelihood ratio (95% CI)	Negative likelihood ratio (95% CI)
KL-6 > 500 U/mL	61 (36–83)	89 (52–100)	5.5 (0.8–36.2)	0.4 (0.2–0.8)
Geographic appearance	89 (65–99)	56 (21–86)	2.0 (0.9–4.2)	0.2 (0.05–0.8)
Geographic appearance and				
KL-6 > 500 U/mL	56 (31–79)	100 (56–100)	NA	0.4 (0.3–0.7)

*CI* confidence interval, *KL-6* Krebs von den Lungen-6

## Discussion

In this study, we demonstrated that the presence of geographic appearance on HRCT and elevated serum KL-6 levels are predictors of DAD in patients with ARDS. ARDS is clinically diagnosed based on the Berlin definition; however, considering these laboratory and radiological findings, it is possible to diagnose ARDS alongside DAD.

Primack et al. first reported that geographic appearance represents HRCT findings in ARDS [12]. According to a later study by Ichimon et al. using autopsy cases of ARDS, postmortem HRCT of the lungs showed geographic appearance with focally spared regions, variably involving several lobules and segments, which revealed findings consistent with the exudative phase of DAD on histopathologic examination [11]. This geographic appearance is currently considered a typical HRCT finding in ARDS [10]. However, geographic appearance is a finding that is evident in various conditions, including pulmonary edema, pulmonary hemorrhage, pneumocystis pneumonia, and acute eosinophilic pneumonia [13]. In fact, our study showed that it has a specificity of only 56%, and therefore the presence of geographic appearance by itself is not sufficient for a DAD diagnosis. However, to the best of our knowledge, this study based on autopsy cases is the first to show an association between geographic appearance and DAD in patients with ARDS.

Our study also suggests that DAD patients had higher KL-6 levels than those without DAD. KL-6 is classified as a human MUC1 mucin protein and is one of the key molecules in interstitial lung diseases [14]. Several previous studies have reported that elevated KL-6 levels are associated with ARDS diagnosis in at-risk populations and ARDS mortality [8, 9]. However, no previous studies have reported an association between DAD and KL-6 levels. As for the mechanism of blood uptake of KL-6, the primary cellular source of KL-6 is regenerating type II pneumocytes in the affected lung; moreover, both the destruction of the alveolar-capillary barrier and the enhancement of alveolar-capillary permeability are necessary for the leakage of KL-6 into the systemic circulation [14]. It seems logical that KL-6 would be higher in patients with DAD than in those with OP or pulmonary hemorrhage, given that endothelial and epithelial damage occur, increasing vascular permeability in the exudative phase of DAD [15]. Renal dysfunction is another known source of KL-6, although there is no known association between serum and urine KL-6 levels [16]. In this study, no significant difference in creatinine levels was observed between the DAD and non-DAD groups, suggesting that the increase in KL-6 levels was due to lung injury. KL-6 was by itself a good predictor of DAD presence, but the combination of geographic appearance and KL-6 may further increase the specificity of the diagnosis.

ARDS is a heterogeneous syndrome. Although DAD is the histopathological hallmark of ARDS, the Berlin definition of ARDS lacks specificity using DAD as the reference standard; a study analyzing 712 autopsied patients showed that the sensitivity and specificity were 89% and 63%, respectively [2]. The Berlin definition has high sensitivity and is an excellent screening tool for diagnosing DAD in an emergency setting. However, high specificity is required for the subsequent accurate diagnosis of DAD. Our method has a high specificity, thus allowing for a more accurate detection of DAD in ARDS patients. The presence or absence of DAD impacts the response to treatment and prognosis, which should be considered in ARDS management. Importantly, the association between the combination of geographic appearance on HRCT and KL-6 levels and DAD should be validated in another larger study to establish a detection method for DAD, which may ensure a better understanding of the pathogenesis and treatment of ARDS.

One of the limitations of our study is that the CT scan and pathological evaluation were not simultaneously carried out. In other words, as the HRCT findings of ARDS change over the clinical course of the disease, the HRCT findings evaluated in this study may not directly reflect the pathological findings at autopsy. However, it is likely that lung pathology was consistent from the time of CT scan to the time of death, and it is clinically important to predict DAD from imaging results at diagnosis. Second, since this study was a single-center retrospective study including only autopsy cases, the sample size was small. Moreover, only patients with ARDS for whom autopsies were performed were included in this study, and the possibility of unintentional selection bias could not be ruled out. Therefore, further validation in clinical trials is required to generalize our results.

## Conclusions

Many patients with DAD on autopsy examination have geographic appearance on HRCT at onset and higher KL-6 levels. The presence of geographic appearance on HRCT combined with KL-6 ≥ 500 is a good predictor of the development of DAD in patients with ARDS.

## Data Availability

The datasets generated and/or analyzed during the current study are not publicly available due to confidentiality and to safeguard accurate data interpretation, but are available from the corresponding author on reasonable request.

## Abbreviations

APACHE: Acute physiology and chronic health evaluation
ARDS: Acute respiratory distress syndrome
CI: Confidence interval
CRP: C-reactive protein
DAD: Diffuse alveolar damage
FiO_2_: Fractional inspired oxygen
HRCT: High-resolution computed tomography
KL-6: Krebs von den Lungen-6
LDH: Lactate dehydrogenase
PaO_2_: Arterial oxygen partial pressure
